# The emerging multifunctional roles of ERAP1, ERAP2 and IRAP between antigen processing and renin-angiotensin system modulation

**DOI:** 10.3389/fimmu.2022.1002375

**Published:** 2022-09-20

**Authors:** Benedetta Mattorre, Valentina Tedeschi, Giorgia Paldino, Maria Teresa Fiorillo, Fabiana Paladini, Rosa Sorrentino

**Affiliations:** Department of Biology and Biotechnology “Charles Darwin”, Sapienza University of Rome, Rome, Italy

**Keywords:** ERAP1, ERAP2, IRAP, Angiotensin, RAS system, hypertension, inflammation, COVID-19

## Abstract

The Endoplasmic Reticulum Aminopeptidase 1 and 2 (ERAP1 and ERAP2) and Insulin Regulated Aminopeptidase (IRAP) are three M1 zinc metalloproteases whose role in antigen processing is the refining of peptidome either in the Endoplasmic reticulum (ERAP1 and ERAP2), or in the endosomes (IRAP). However, other novel and distinct functions are emerging. Here, we focus specifically on ERAP2. This gene has a peculiar evolutionary history, being absent in rodents and undergoing in humans to a balanced selection of two haplotypes, one of which not expressing the full length ERAP2. These observations suggest that its role in antigen presentation is not essential. An additional, less investigated role is in the regulation of the Renin Angiotensin System (RAS). ERAP1 and ERAP2 cleave Angiotensin II (Ang II) into Ang III and IV, which counteract the action of Ang II whereas IRAP is itself the receptor for Ang IV. We have recently reported that macrophages, independently from the haplotype, express and release a N-terminus ERAP2 “short” form which directly binds IRAP and the two molecules are co-expressed in the endosomes and on the cell membrane. This new evidence suggests that the maintenance of the ERAP2 gene in humans could be due to its activity in the regulation of the RAS system, possibly as an Ang IV agonist. Its role in the immune-mediated diseases as well as in disorders more specifically related to an imbalance of the RAS system, including hypertension, pre-eclampsia but also viral infections such as COVID-19, is discussed here.

## ERAP1, ERAP2 and IRAP in multifactorial diseases: genetic associations

ERAP1 and ERAP2 belong to the subfamily of oxytocinases, a group of M1 zinc-metalloproteases. These enzymes share 50% identity in their amino acid sequence and their more investigated role is in the N-terminal processing of proteasome-derived peptides with an unsuitable length for Human Leukocyte Antigen (HLA)-I molecules (1, 2). In fact, cytosolic peptides are transported by Transporter Associated with Antigen Processing (TAP) molecules into the ER where they are further trimmed by the ERAPs. The electrostatic potential of ERAP1 and ERAP2 is different, meaning that these two enzymes have different affinities towards the peptide substrate. Indeed, ERAP1 cleaves N-terminal hydrophobic amino acids while ERAP2 prefers residues with positively charged side chains (3, 4). Furthermore, ERAP1 binds longer peptides (8-16 aa) than ERAP2. Cleavage of longer peptides probably requires the concerted action of both ERAPs and, accordingly, it has been found that ERAP1 and ERAP2 co-localize *in vivo* and form heterodimeric complexes (2). Interestingly however, while ERAP1 is expressed in humans as well as in rodents, ERAP2 gene is missing in rodents and, although present in the human genome, is not expressed as full-length protein in about 25% of the human population, suggesting that its role is, at any rate, dispensable (5, 6).

In the human genome, ERAP1 and ERAP2 genes lie on chromosome 5q21 in the opposite orientation contiguously to Leucyl and Cystinyl Aminopeptidase (LNPEP) gene and share sequence and function as aminopeptidases. The LNPEP gene encodes for an endosomal and membrane aminopeptidase also named Insulin-Regulated Aminopeptidase (IRAP), which cleaves predominantly before cysteine and leucine. IRAP, together with the ERAPs, belongs to the oxytocinase family and shares 43 and 49% identity with ERAP1 and ERAP2, respectively. This aminopeptidase is involved in the cross-presentation, in particular refining cross-presented peptides in the endosomes of Dendritic Cells (DC) (7).

Several genetic studies highlight a considerable involvement of the three aminopeptidases in Immune-Mediated Diseases (IMD) as well as in several other complex pathologies. In genomic investigations on a worldwide scale, i.e. Genome Wide Association Studies (GWAS), authors have identified in the coding and non-coding regions, Single Nucleotide Polymorphisms (SNPs) associated with Ankylosing Spondylitis (AS), Behçet’s disease (BD), Psoriasis (Ps), Inflammatory Bowel Disease (IBD), Juvenile Idiopathic Arthritis (JIA), Birdshot chorioretinopathy (BSCR), Type I Diabetes (T1D) and Multiple Sclerosis (MS) (8, 9). In particular, SNPs rs30187 (K528R) and rs27044 (Q730E) have been found to be associated with AS (10, 11). The K528R amino acid substitution appears to determine, both *in vitro* and *in vivo*, a defect in the catalytic cycle with an alteration of the peptide cleavage activity, while the Q730E substitution seems to have an effect both in the specificity of the trimming as well as in the length preference of the peptide (12–14). It is interesting to observe that some protective allelic variants in a specific HLA-associated disease, may instead be risk variants to other chronic diseases associated or not with HLA alleles. For example, in Ankylosing Spondylitis, variant G in rs30187 (R528) appears to be protective in subjects positive for HLA-B*27, the major susceptibility gene in AS, whereas it is a risk factor for Behçet’s disease in carriers of HLA-B*51, the allele more strongly associated with BD (15). Notably, in both diseases, an epistatic mechanism between ERAP1 and the relevant HLA-I genes, HLA-B*27 and HLA-B*51, has been demonstrated, pointing to an aberrant processing of antigenic peptides as cause of the disease (12, 16). It must be mentioned however, that a same variant (R528) is associated with hypertension, due to a reduced inactivation of AngII and clearly indicating that an additional/alternative mechanism of susceptibility can exist (17, 18). ERAP2 has also been implicated in different HLA-class I-associated diseases such as AS and BSCR. In both cases, the absence of the full length ERAP2 is protective (18, 19). Since BSCR patients are virtually 100% HLA-A*29 positive, an epistatic mechanism could not be evaluated (20). In the case of AS however, the risk conferred by ERAP2 is independent of HLA-B*27 (21) suggesting that the process by which ERAP2 affects the disease pathogenesis could be different from antigen processing.

Interestingly, the expression of ERAP1 and ERAP2 has been shown to be concerted by a polymorphism located in the ERAP2 promoter region, SNP rs75862629, where the presence of the major allele A allows the binding of a transcription factor which is inhibited by the alternative minor allele G. In the latter case, a decrease in the ERAP2 expression is coupled with a higher expression of ERAP1, thus indicating a coordinated quantitative regulation of the two ERAP genes (22, 23).

Another polymorphism specifically regulates the expression of ERAP2: the SNP rs2248374, located within the 5’ splicing site of exon 10, determines whether ERAP2 full length will be expressed. In a recent study indeed, this polymorphism is suggested to be under balancing selection, displaying two haplotypes of similar frequencies (haplotypes A and B at 0.44 and 0.56 frequencies, respectively) (5). In the haplotype A, the SNP at position rs2248374 is an adenine (A) whereas in the B haplotype is a guanine (G). These two variants undergo a different splicing mechanism, which leads to the production of two different mRNAs: haplotype A gives rise to a mRNA that encodes the canonical protein of 960 aa, whereas haplotype B displays a premature stop codon within exon 10 that causes Non-sense Mediated Decay (NMD) of the coding RNA (5). Thus, homozygotes for haplotype A or AB heterozygotes can produce the canonical protein. Accordingly, ~3/4 of the population express a functional ERAP2 while the remaining ~25% does not (5). There are, however, exceptions. For instance some transformed cells, although being genotyped as G/G at rs2248374, have been shown to express full length ERAP2 (24, 25), suggesting that the NMD can be overcome under some circumstances. Interestingly, an ortholog of the ERAP2 gene is lacking in several other species among which rodents (6). This, together with the lack of the ERAP2 protein in 25% of humans, suggests that the ERAP2 function is redundant. As for ERAP1 and ERAP2, IRAP polymorphisms are also associated with several disorders such as Psoriasis, Breast cancer, Type I Diabetes, Alzheimer’s disease and Septic Shock (26–29). In particular, the LNPEP single nucleotide variation at rs4869317, located in the regulatory region of the gene, has a high correlation with the vasopressinase activity and leads to an increased mortality caused by Septic Shock (26). The missense LNPEP variant A/G at rs2303138 encoding for a threonine (ACA) instead of alanine (GCA), appears to be associated with Psoriasis providing validity to the hypothesis that renin-angiotensin system can be involved in the pathology (27). In addition, patients carrying this variant have a greater susceptibility to systemic comorbidities such as cardiovascular diseases, metabolic syndrome, diabetes, pathological conditions that could be due to the role played by IRAP in the process of glucose uptake (30), vasopressin clearance and sodium regulation (31, 32).

## Functions of the three aminopeptidases: ERAP1, ERAP2 and IRAP beyond antigen processing

Besides antigen processing functions, the three aminopeptidases appear to play an essential role in many physiological processes relevant for the maintenance of cellular homeostasis (6). Accordingly, ERAP1 is also involved in the shedding of the extracellular portion of the cytokine receptors, such as Interleukin (IL)-1RII, IL-6R and Tumor Necrosis Factor (TNF)-R. This process generates the soluble forms that are released at the extracellular level and appears to be involved in the regulation of innate immunity and the inflammatory response (33). ERAP1 has also been described as an important regulator of postnatal neo-angiogenesis induced by the Vascular Endothelial Growth Factor (VEGF), a physiological process that leads to the production of new blood vessels from pre-existing structures. In fact, several *in vitro* and *in vivo* functional studies have shown that, in pre-existing endothelial cells, an inhibition of ERAP1 leads to an altered cell proliferation (34). ERAP1 also appears to have a role in innate immunity, since Interferon (IFN)-γ and bacterial Lipopolysaccharide (LPS)-stimulated macrophages release a secreted form of ERAP1, which, in turn, induces a Fc receptor-dependent phagocytic activity (35).

Besides being localized in the endosomal vesicles, where it plays a role in cross-presentation, IRAP is also secreted as a soluble form in the maternal serum during pregnancy (36). Moreover, IRAP might move to the cell membrane generating a type II integral membrane glycoprotein upon the addition of a N-terminal cytoplasmic domain. The cytosolic portion of IRAP appears to interact with a GTPase Activating Protein (GAP) that prevents the translocation of glucose transporter 4 (GLUT4) until glucose uptake in insulin responding cells promotes its intracellular trafficking (37). Studies carried out *in vivo* in mouse models have confirmed the functional interaction between GLUT4 and IRAP, since the knockout of either partner appears to affect the stability of the other (38). Very recently, a direct interaction in the endosomes between IRAP and the T Cell Receptor (TCR) has been demonstrated and, most interestingly, IRAP deletion compromises the TCR-CD3ζ signaling (39). In addition, given its expression in the brain, heart, kidney, adrenal gland and blood vessels, IRAP appears to contribute to additional, still not well-defined functions, aimed to maintain a physiological balance including proper blood flow, neuroprotection, synaptogenesis, long-term enhancement and memory consolidation (40, 41).

Among the three aminopeptidases, ERAP2 is the one for which additional role have not been well defined as yet. However, its role in the RAS system is now generally accepted (4, 42, 43).

## ERAP1, ERAP2 and IRAP in the renin-angiotensin system

**Figure 1:**
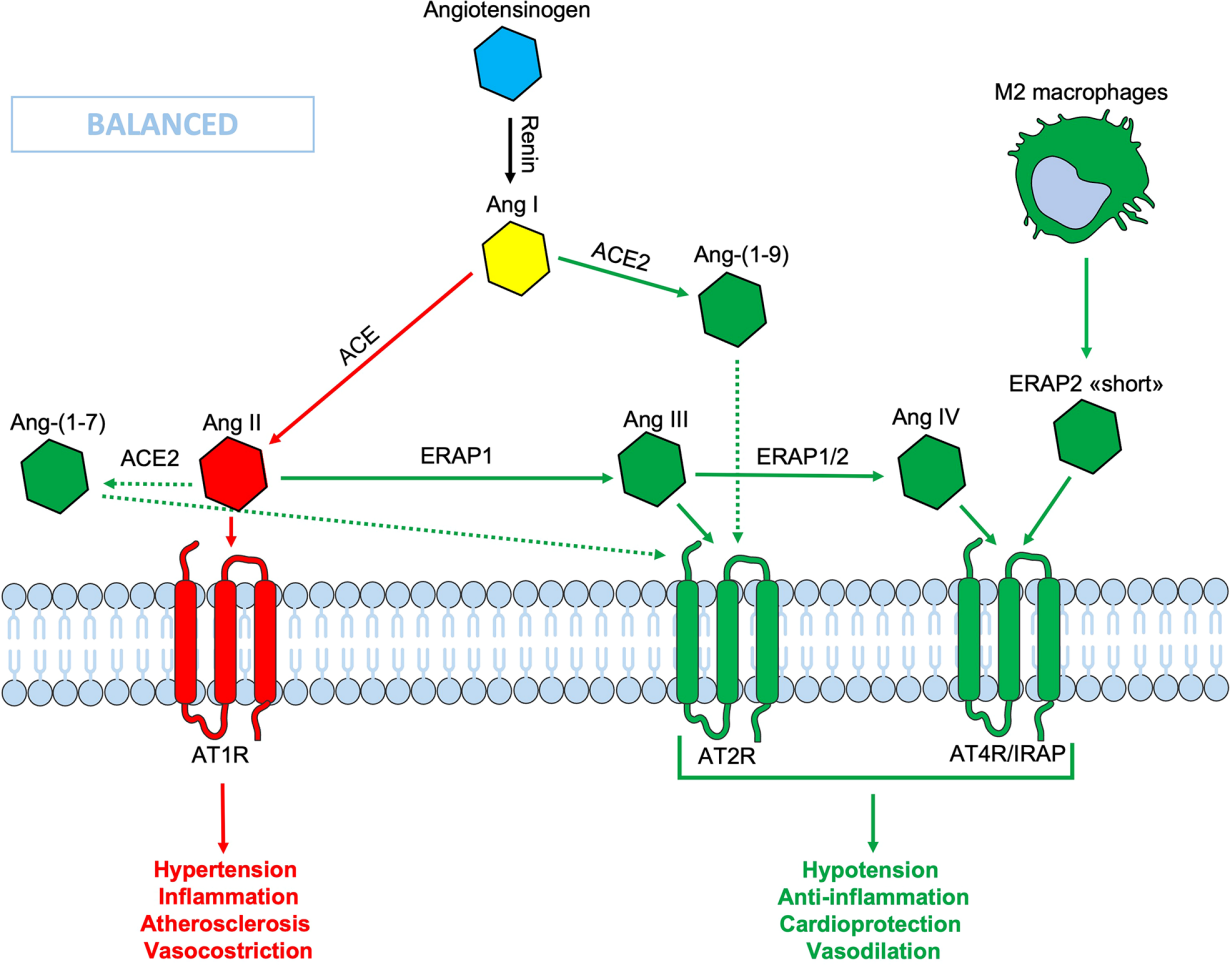
Schematic representation of the Renin-Angiotensin System (RAS). In the classic ACE/Ang II/AT1R axis (red lines), the Ang II binds its AT1R receptor, causing hypertension, inflammation, vasoconstriction and fibrosis. Thanks to the ACE2/Ang-(1-9)/AT2R-AT4R axis (green lines) which counteracts the effects of the opposite axis, the balance is maintained. In this context the ERAPs, through their cutting activity, contribute to the homeostasis. The binding of ERAP2 “short” form to AT4R/IRAP could contribute to maintain the RAS system balance, even when the ERAPs are defective. Ang, angiotensin; ACE, angiotensin converting enzyme; AT1R, angiotensin type 1 receptor; AT2R, angiotensin type 2 receptor; AT4R, angiotensin type 4 receptor; ERAP1, Endoplasmic Reticulum Aminopeptidase 1; ERAP2, Endoplasmic Reticulum Aminopeptidase 2; IRAP, Insulin-Regulated Aminopeptidase.

In the RAS system, plasma renin converts angiotensinogen to Ang I which, in turn, is converted to Ang II by the Angiotensin Converting Enzyme (ACE) (EC.3.4.15.1) located on the surface of vascular endothelial cells (44). Ang II is a potent vasoconstrictor agent that causes the narrowing of blood vessels and an increase in blood pressure (45). Ang II levels can also be regulated by the Angiotensin Converting Enzyme 2 (ACE2) (EC 3.4.17.23), which leads to the production of the vasodilating heptapeptide Ang- (1–7), and by other aminopeptidases (46, 47). Indeed, *in vitro* studies have shown that ERAP1 catalyzes the conversion of Ang II into Ang III and IV, while ERAP2 converts Ang III to Ang IV (**Figure 1**) (48, 49). In particular, ERAP1 has been found to shuttle between the intracellular and the extracellular compartment where it acts as blood pressure regulator operating on Ang II (50). The interaction of Ang III and Ang IV with their respective receptors, AT2R and AT4R/IRAP, leads to a reduction in inflammation and a higher level of nitric oxide (NO) resulting in the lowering of blood pressure (**Figure 1**). Recent studies have shown how the RAS system and Ang II in particular, can play an important role in local inflammatory processes, autoimmunity and cellular aging (51). It has been shown that many stimuli relevant to cardiovascular diseases, including cytokines (IL-6 and TNFα), growth factors, Ang II and signals caused by ischemic stress (NO and oxygen free radicals), are connected to the NF (Nuclear Factor)-κB pathway (52). Notably, ERAP2 has been found associated with pre-eclampsia, a disease that is characterized by an increased blood pressure during pregnancy (53). However, the mechanism by which genetic variants influence the risk of pre-eclampsia remains to be elucidated. More recently, ERAP2 gene has also been identified as a risk factor for death by COVID-19 (54). IRAP cleaves vasopressin and oxytocin and catalyzes the final step in the conversion of angiotensinogen to angiotensin IV (AT4) and, indeed, it is also known as angiotensin IV receptor (AT4R) due to its high affinity binding of angiotensin IV. The AT4R is highly expressed in the heart, skeletal muscles, kidneys, small intestine, placenta and blood vessels and alternative splicings result in multiple transcript variants encoding different isoforms. Interestingly, placental AT4R is upregulated during early pregnancy implicating its role in the migration of extravillous trophoblasts (EVT) (55).

## ERAP2 “short” form: A key role in RAS balance?

ERAP1 and ERAP2 can decrease the circulating Ang II levels, converting it into Ang III, the main ligand for the Angiotensin Type 2 Receptor (AT2R) and into Ang IV, the main ligand of AT4R/IRAP (42, 56, 57). The binding of both Ang III and IV with their respective receptors has opposite effects in respect to the Ang II when bound to AT1R, thus contributing to cellular and tissue homeostasis and to the RAS system balance (**Figure 1**). A less efficient cleavage of Ang II by the ERAPs, due to polymorphic variations, could lead to an imbalance of the RAS system in favor of an inflammatory state, NO production inhibition and hypertension, atherosclerosis and vasoconstriction (**Figure 1**). A new form of ERAP2, that is expressed and secreted regardless of gene variations by M2 macrophages, but not by monocytes or other blood mononuclear cells, has been recently described by our group (25). This “short” form, most likely generated by an autocatalytic process in the endosomes, has a molecular weight of about 55 KDa and maintains the ERAP2 N-terminal end. It must be pointed out that this is different from the ERAP2/iso3 variant of similar MW, an alternative spliced isoform described as triggered by flu-infection and other pathogens including HIV (Human Immunodeficiency Virus), SARS-CoV-2, CMV (Cytomegalovirus) and LPS-positive bacteria and which, however, does not possess enzymatic activity *per se* (58, 59). Most interestingly, the “short” ERAP2 directly binds IRAP and it is co-expressed in endosomes as well as on the cell membrane (25). This is a novel finding that connects for the first time the two aminopeptidases, ERAP2 and IRAP, which can eventually co-operate in the RAS system, although at this stage their enzymatic activity has to be proven. Here, we consider the hypothesis that the release of this “short” ERAP2 by M2 macrophages and its binding to the Ang IV receptor, can contribute to the balance of the RAS system, possibly working as an agonist for IRAP. In this way, it could contribute to the anti-inflammatory activity exerted locally by the M2 macrophage and, maybe, help to counteract the hypertension, inflammation, pro-fibrotic activity and vascular permeability (**Figure 1**).

## Could the “short” ERAP2/AT4R axis play a role in the protection against severe COVID-19 infection?

**Figure 2:**
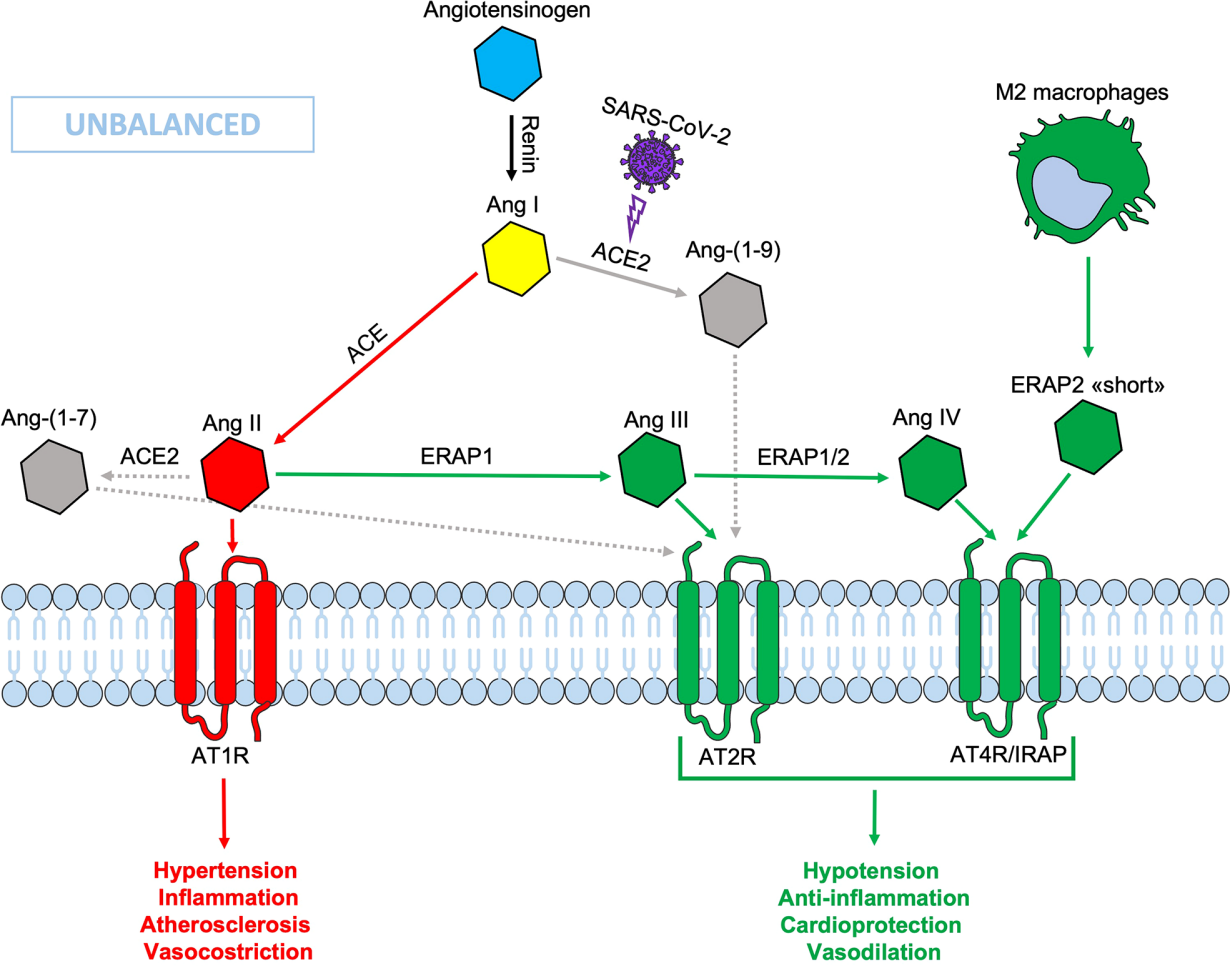
Schematic representation of the Renin-Angiotensin System (RAS) during SARS-CoV-2 infection. The down-modulation of ACE 2 induced by viral infection generates an imbalance in the RAS system, resulting in an increased Ang II circulating levels. Non-functional elements of the axis are marked in gray. Ang, angiotensin; ACE, angiotensin converting enzyme; AT1R, angiotensin type 1 receptor; AT2R, angiotensin type 2 receptor; AT4R, angiotensin type 4 receptor; ERAP1, Endoplasmic Reticulum Aminopeptidase 1; ERAP2, Endoplasmic Reticulum Aminopeptidase 2; IRAP, Insulin-Regulated Aminopeptidase.

**Figure 3:**
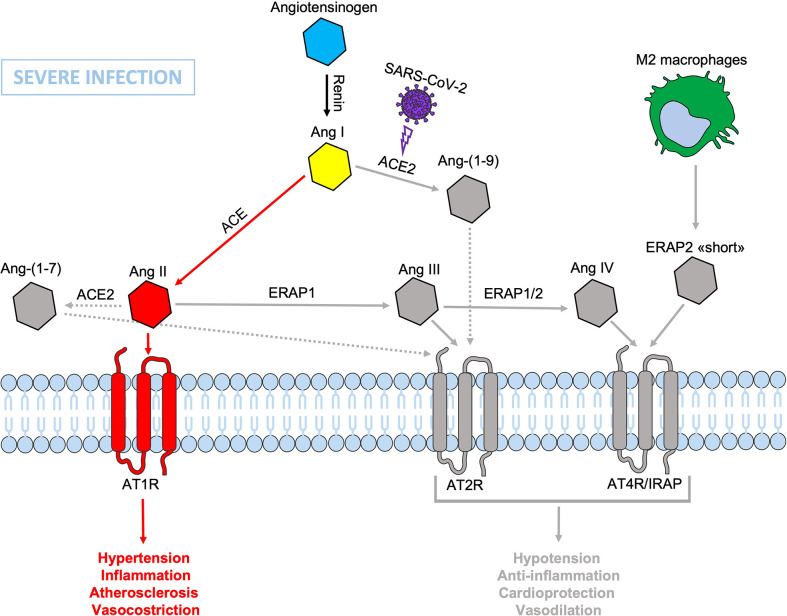
Schematic representation of the Renin-Angiotensin System (RAS) during SARS-CoV-2 infection. The presence of dysfunctional ERAP1 and ERAP2 would be disadvantageous and/or even increase the severity of COVID-19 disease course in individuals carrying also genetic variants inducing defects in the binding between ERAP2 “short” form and IRAP/AT4R leading to a lack or reduced signal transduction that accentuates the imbalance in favor of hypertension, vasoconstriction, inflammation, increased vascular permeability and atherosclerosis resulting in impaired blood flow, all factors known to be associated with lung injury and cardiac damage. Non-functional elements of the axis are marked in gray. Ang, angiotensin; ACE, angiotensin converting enzyme; AT1R, angiotensin type 1 receptor; AT2R, angiotensin type 2 receptor; AT4R, angiotensin type 4 receptor; ERAP1, Endoplasmic Reticulum Aminopeptidase 1; ERAP2, Endoplasmic Reticulum Aminopeptidase 2; IRAP, Insulin-Regulated Aminopeptidase.

During SARS-CoV-2 infection, the surface expression of ACE2 is reduced by endocytosis and proteolytic cleavage after viral binding (60). The reduction of ACE2 generates an imbalance in the RAS system: on one side an increased level of circulating Ang II, on the other a decreased signaling by the ACE2-derived peptides (Ang 1-7) resulting in hypertension, inflammation and lung damage (**Figure 2**) (61). Several studies have shown an increase in the circulating Ang II in patients with COVID-19 compared to healthy controls, suggesting that the reduction of ACE2 expression, causing a RAS imbalance, can lead to an uncontrolled inflammatory response with multi-organ consequences (61, 62). Accordingly, new therapeutic strategies based on the recombinant human ACE2 (rhACE2 or ACE2-homologs) could be beneficial to contrast the effects of COVID-19 infection and the imbalance caused by SARS-CoV-2 in the RAS system (63). The ERAP1 and ERAP2 cleavage activities of the circulating Ang II could be crucial in restraining the effects of ACE2 downregulation (**Figure 3**). In this context, ERAP1 and ERAP2 variants leading to a reduced cleavage of Ang II into Ang III and Ang IV, may contribute to a more severe outcome of COVID-19 infection by unbalancing the RAS system and resulting in hypertension, vasoconstriction and inflammation with consequent impairment of blood flow (64) (**Figure 3**). It is possible that this “short” ERAP2, once secreted by M2 macrophages, may play a role by directly binding IRAP. In fact, these aminopeptidases have a two-fold task: lowering the level of Ang II through their catalytic activity and counterbalancing the effects of Ang II through the binding to AT4R/IRAP by the ERAP2 “short” form (55KDa), and thus favoring cellular and tissues homeostasis.

## Conclusions

Our previous analysis of the expression of the three aminopeptidases genes, ERAP1, ERAP2 and IRAP, along the evolutionary zoological scale, had led us to formulate the hypothesis that these three genes have acquired their role in antigen presentation later on in the evolution and that their primary and more ancient function is the regulation of the RAS system. Along this line, ERAP2 gene is singular since it has been lost by rodents while maintained in humans through a balanced selection allowing its full expression in only ~25% of the population and its absence in another ~25%. Our finding that an ERAP2 “short” form is expressed by M2-macrophages independently from the gene haplotype and therefore by 100% of the population, has important implications. Indeed, this “short” form can bind AT4R/IRAP supporting the idea that the maintenance of its expression in humans is due to its role in the RAS system, eventually working as agonist of AT4R/IRAP and playing a role in dumping inflammation in many different circumstances. This is supported by the observation that the full length ERAP2 is clearly dispensable and sometimes detrimental as suggested by the fact that its expression is tuned down by a mechanism of allelic exclusion and that the lack of its expression as full-length protein has a protective effect in several immune-mediated diseases.

## Author contributions

Conceptualization: BM, FP, and RS Writing—Original Draft Preparation: BM and RS, Writing—Review and Editing: BM, MF, VT, GP, FP, RS. Funding Acquisition, RS and MF. All authors have read and agreed to the published version of the manuscript.

## Funding

This study was supported by Fondazione Ceschina (http://fondazioneceschina.org) and by Sapienza, University of Rome (Progetti di Ateneo) to RS and MF.

## Conflict of interest

The authors declare that the research was conducted in the absence of any commercial or financial relationships that could be construed as a potential conflict of interest.

## Publisher’s note

All claims expressed in this article are solely those of the authors and do not necessarily represent those of their affiliated organizations, or those of the publisher, the editors and the reviewers. Any product that may be evaluated in this article, or claim that may be made by its manufacturer, is not guaranteed or endorsed by the publisher.
